# Endogenous Price Bubbles in a Multi-Agent System of the Housing Market

**DOI:** 10.1371/journal.pone.0129070

**Published:** 2015-06-24

**Authors:** Roy Kouwenberg, Remco C. J. Zwinkels

**Affiliations:** 1 College of Management, Mahidol University, Bangkok, Thailand; 2 Finance Department, VU University, Amsterdam, The Netherlands; 3 Tinbergen Institute, Amsterdam, The Netherlands; Uppsala University, SWEDEN

## Abstract

Economic history shows a large number of boom-bust cycles, with the U.S. real estate market as one of the latest examples. Classical economic models have not been able to provide a full explanation for this type of market dynamics. Therefore, we analyze home prices in the U.S. using an alternative approach, a multi-agent complex system. Instead of the classical assumptions of agent rationality and market efficiency, agents in the model are heterogeneous, adaptive, and boundedly rational. We estimate the multi-agent system with historical house prices for the U.S. market. The model fits the data well and a deterministic version of the model can endogenously produce boom-and-bust cycles on the basis of the estimated coefficients. This implies that trading between agents themselves can create major price swings in absence of fundamental news.

## Introduction

The global financial crisis and subsequent sovereign debt crisis have illustrated that standard economic models are not capable of capturing the complex price dynamics observed in real markets. The core principles of standard economic models are agent rationality and market efficiency. With these assumptions, however, the models are incapable of explaining boom and bust cycles. Former president of the European Central Bank, Jean Claude Trichet, nicely summarized it as follows: “In the face of the crisis, we felt abandoned by conventional tools. […] The atomistic, optimizing agents underlying existing models do not capture behavior during a crisis period. We need to deal better with heterogeneity across agents and the interaction among those heterogeneous agents.”

Multi-agent complex systems offer an important and promising alternative modeling approach. Originating from the econophysics literature, this framework does not assume that all agents are rational, but rather that they are heterogeneous and follow different rules to predict future prices [1,2]. Typically a group of agents in these models called 'fundamentalists' believe in market efficiency and expect the price to revert to the present value of future payoffs. However, a second group of agents called 'chartists', simply expect past price trends to continue. Using analytical and simulation methods, multi-agent models have been shown to replicate well-known characteristics of market returns better than traditional economic models [3].

In this paper we develop and estimate a simple multi-agent model for the U.S. housing market. The development of better models for the housing market is of high importance, in the light of the failure of financial institutions and regulators to predict the house price collapse that triggered the global financial crisis in 2008–2009. The housing market is more vulnerable to inefficiencies than other markets due to lack of effective short selling mechanisms that prevent bearish investors from participating. Furthermore, the heterogeneity in housing stock as well as the heterogeneity in market participants prevents standard arbitrage processes from functioning properly. Our main result is that the interaction between agents in the model can generate boom-bust cycles endogenously, even in the absence of underlying fundamental news. Agents in the model can switch between the fundamentalist and chartist forecasting rules, depending on the rules' recent prediction performance. Precisely this feature allows the market to be driven by chartists when a price bubble builds up, but dominated by fundamentalists during the eventual burst [4,5]. In the companion article [6], we further show that the econometric model derived from this multi-agent system delivers better out-of-sample price forecasts for the U.S. housing market than standard models.

Our paper contributes to the recent theoretical and empirical literature on agent-based models for the housing market [7–12]. The main contribution is that we show that a model with chartists and fundamentalists endogenously produces boom and bust cycles, on the basis of parameters values estimated with U.S. housing market data covering the period 1960–2014. Our model is adapted from [7,8], who introduce an agent-based model for the housing market. [7,8] show that for certain parameterizations the model can generate endogenous boom-and-bust patterns, but they did not estimate the parameters with historical data like we do here. In companion paper [6], we estimate a smooth transition model for the U.S. housing market inspired by the agent-based literature and focus on its out-of-sample prediction performance. In this paper we provide a behavioural foundation for the econometric model used by [6] to forecast U.S. house prices.

[9] propose an agent-based housing market model where agents have different beliefs about the fundamental value. [10] build an agent-based model for the housing market in the Washington D.C. area using data on 2.2 million homeowners; the model fits actual house prices in the period 1997–2010 well. [11] estimate a heterogeneous agent model with fundamentalists and chartists for the housing market in eight countries. The main difference is that in our model the house price adjustment is based on excess demand, whereas in [11] price changes are derived from a temporary equilibrium pricing model. [12] estimate a heterogeneous agent model similar to ours using 350 years of data on house prices in Amsterdam, but using consumer prices instead of rent data to estimate fundamental house values.

## Methods and Data

### A simple agent based model

Following [7], the market in our model is populated by three types of agents: consumers, constructors and investors. Consumers and investors are on the demand side of the market, while constructors are on the supply side. Consumers buy houses for the sole purpose of shelter. We assume that the flow of aggregate consumer demand for housing ($D_{t}^{C}$) depends on the value of the house price index at time *t*:

$$D_{t+1}^{C}=a+bP_{t},$$ (1)

where *t* is time measured in quarters and *P*_*t*_ is the logarithm of the real house price index at time *t*.

The investors in our model are only interested in short-term capital gains, and not motivated by long-term rent income. Investors choose among two forecasting rules for determining the expected return *E*(*R*_*t*+1_), called fundamentalist and chartist. The return *R*_*t*+1_ is defined as the real log-price change *P*_*t*+1_-*P*_*t*_. The first rule, fundamentalist, is based on the expectation of mean reversion of the market price towards the long-term fundamental value.

$$E_{t}^{f}(R_{t+1})=\alpha (P_{t}-F_{t})$$ (2)

in which *F*_*t*_ is the log real fundamental price and *α*<0 the speed of mean reversion expected by the fundamentalist investors.

We assume that all investors are mean-variance maximizers, with the same level of risk aversion (*η*) and with the same beliefs about the conditional variance of housing returns (*σ* ^2^). Under these conditions [5] show that the speculative demand of investors ($D_{t}^{f}$) is a linear function of the expected return:

$$D_{t+1}^{f}=\frac{1}{\eta \sigma^{2}}E_{t}^{f}(R_{t+1})=k\alpha (P_{t}-F_{t}),$$ (3)

in which *η>0* represent the investors' risk aversion parameter, *σ* ^2^>0 is the constant variance of housing returns and *k* = 1/*ησ* ^2^>0.

The second rule, which we call chartist, takes advantage of positive autocorrelation in housing returns, documented by [13]. Chartist expectations are given by

$$E_{t}^{c}(R_{t+1})=\beta \left(\sum_{l=1}^{L}R_{t-l+1}\right)$$ (4)

in which *β*>0 is the extrapolation parameter, and *L*>0 is a positive integer indicating the number of lags. Chartists simply expect past price changes to continue in the future, without considering the fundamental value. Given the assumption of mean-variance preferences, the speculative demand of chartists ($D_{t}^{c}$) is a linear function of past housing returns:

$$D_{t+1}^{c}=\frac{1}{\eta \sigma^{2}}E_{t}^{c}(R_{t+1})=k\beta \left(\sum_{l=1}^{L}R_{t-l+1}\right).$$ (5)

Whereas agents in the model of [7] switch based on the distance between price and fundamental value, investors in our model switch between the two forecasting rules depending on their recent prediction performance. For this purpose we use a logit switching rule, as introduced by [14] and applied in [4,5], such that the weight of fundamentalists *W*_*t*_ϵ<0,1> is given by

$$W_{t}={\left(1+\text{exp}\left[\gamma \left(\frac{\pi_{t}^{f}-\pi_{t}^{c}}{\pi_{t}^{f}+\pi_{t}^{c}}\right)\right]\right)}^{-1},\;$$ (6)

and the chartist weight is equal to (1-*W*_*t*_), in which $\pi_{t}^{f}$ and $\pi_{t}^{c}$ are the observed forecast errors over the recent past of the fundamentalist and chartist rules at time *t*, respectively. The parameter *γ*>0 captures the sensitivity of investors to differences in forecast errors between the two rules. Higher values of $\pi_{t}^{f}$ and $\pi_{t}^{c}$ imply bigger forecast errors, and a positive value of *γ* then causes investors to give more weight to the better performing rule.

Strategy performance, measured by $\pi_{t}^{f}$ and $\pi_{t}^{c}$, is based on the observed absolute forecast errors of the fundamentalist and chartist rules in the previous *K* periods. That is,

$$\pi_{t}^{f}=\sum_{k=1}^{K}\left|E_{t-k}^{f}(R_{t-k+1})-R_{t-k+1}\right|,$$ (7)

$$\pi_{t}^{c}=\sum_{k=1}^{K}\left|E_{t-k}^{c}(R_{t-k+1})-R_{t-k+1}\right|.$$ (8)

Total demand by investors is then the weighted average demand of fundamentalists and chartists, and can be written as follows:

$$D_{t+1}^{I}=W_{t}D_{t+1}^{f}+(1-W_{t})D_{t+1}^{c}.$$ (9)

Apart from demand for housing by consumers and investors, constructors build new residential structures and sell them in the market. The new supply by constructers (*S*_*t*_) depends positively on the value of the house price index at time *t*:

$$S_{t+1}=c+dP_{t},$$ (10)

in which *c*>0 and *d*>0.

We assume that the overall change in the log real house price depends linearly on excess demand plus a random noise term *ε*_*t*_, which can be thought of as the impact of pure noise traders:

$$P_{t+1}-P_{t}=f(D_{t+1}^{C}+D_{t+1}^{I}-S_{t+1})+\varepsilon_{t+1},$$ (11)

where *f*>0 is a positive reaction parameter. Filling in the different elements from Eqs (1) to (10) into (11) yields the following equation for the house price dynamics

$$R_{t+1}=f\left((a-c)+(b-d)P_{t}+W_{t}k\alpha (P_{t}-F_{t})+(1-W_{t})k\beta \sum_{l=1}^{L}R_{t-l+1}\right)+\varepsilon_{t+1}.$$ (12)

Without loss of generality, we can assume that *f* = 1 and *k* = 1, because the utility function is invariant to a positive linear transformation, such that the empirical model can be written as

$$\left\{ \begin{array}{l} R_{t+1}=c'+d'P_{t}+W_{t}\alpha (P_{t}-F_{t})+(1-W_{t})\beta \sum_{l=1}^{L}R_{t-l+1}+\varepsilon_{t+1}\\ W_{t}={\left(1+\text{exp}\left[\gamma \left(\frac{\pi_{t}^{f}-\pi_{t}^{c}}{\pi_{t}^{f}+\pi_{t}^{c}}\right)\right]\right)}^{-1}\\ \pi_{t}^{f}=\sum_{k=1}^{K}\left|\alpha (P_{t-k}-F_{t-k})-R_{t-k+1}\right|\\ \pi_{t}^{c}=\sum_{k=1}^{K}\left|\beta \sum_{l=1}^{L}R_{t-k-l+1}-R_{t-k+1}\right| \end{array}\right.$$ (13)

in which the intercept is given by *c*′ = (*a*-*c*) and *d*′ = (*b*-*d*).

### Data and Estimation Method

#### U.S. house price data and rent-based fundamental values

Estimation of the model coefficients requires a reliable series of historical house price values (*P*_*t*_) and information that can used to estimate the fundamental house value (*F*_*t*_). Following [6], we use quarterly time-series data on prices and rents for the aggregate stock of owner-occupied housing in the United States developed by [15], and made available by the Lincoln Institute of Land Policy. The data is located at “Land and Property Values in the U.S.”, Lincoln Institute of Land Policy, http://www.lincolninst.edu/resources/. An Excel file with the U.S. house price data and the rent data can also be directly accessed at: http://www.lincolninst.edu/subcenters/land-values/rent-price-ratio.asp. The house price data is based on the S&P/Case-Shiller U.S. National Home Price Index. The data cover the period from 1960Q1 through 2014Q1, the most recently available values at the time of writing.

**Fig 1:**
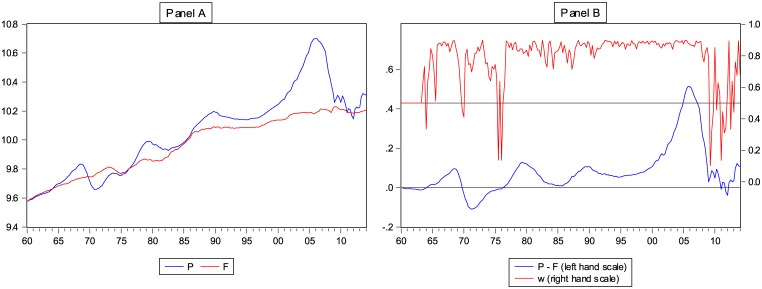
U.S. House Price Index and Fundamental Value Estimate. Panel A of Fig 1 displays the log-real U.S. house price index *P*_*t*_ and the log-real fundamental value estimate *F*_*t*_. The upper part of Panel B displays the weight *W*_*t*_, the fraction of investors using the fundamentalist forecasting rule (right axis). The lower part of Panel B (left axis), displays *P*_*t-*_ *F*_*t*_, the difference between the house price and its fundamental value.

In [6] we construct a rent-based fundamental value index for the aggregate U.S. housing market, using these data. Briefly, the fundamental value estimate is the present value of all future expected rent income, under the assumption that the growth rate of rents is constant. Fig 1 shows the resulting log fundamental price, and the actual log U.S. house price index for comparison, in the period from 1960Q1 until 2014Q1.

Panel A of Fig 1 shows that the actual house price tends to oscillate around the rent-based fundamental value estimate. The recent boom and crash in U.S. house prices can be easily identified on the right end of Fig 1, and resembles a large price bubble. In the first quarter of 2006 the overvaluation of the U.S. housing market reached its maximum, when the log house price was 48% above its fundamental value. This was an unprecedented situation, since the misalignment had never exceeded the 10% mark before.

#### Model estimation method

Model estimation is done similarly as in Kouwenberg and Zwinkels [6], that is, by writing Eq (13) as single non-linear equation and applying maximum likelihood estimation. Starting values for all coefficients but *γ* are chosen by first estimating the static non-switching version of the model with *γ = 0*. A starting value for *γ* is then found by applying a grid-search over a range of reasonable values and selecting the best fitting one. The values of the lag parameters *K* and *L* are chosen as follows: we consider all integer values of *K* and *L* between one and twelve, and select the model with the best fit based on log-likelihood.

## Results

### Model estimation results

Table 1 presents the in-sample estimation results, estimated using quarterly U.S. house price data from 1960Q1 until 2014Q1. The optimal lags parameters are *K* = 1 and *L* = 4.

**Table 1:** Multi-Agent Model Estimation Results.

*Coefficient*	*c*′	*d*′	*α*	*β*	*γ*	Observations
Estimate	-0.2307***	0.0236***	-0.6329***	0.3032***	2.1818***	204
(Std. error)	(0.0704)	(0.0070)	(0.0757)	(0.0212)	(0.1952)	

The table shows the estimated coefficients of the heterogeneous agent model in Eq (13), using quarterly data on U.S. house prices and rents from 1960Q1 to 2014Q1. Robust standard errors are shown in parentheses below the estimates.

*, **, *** denotes significance at the 10%, 5%, and 1% level.

The coefficients for the fundamentalist and chartist rules in Table 1 are significant and have the expected signs. The negative sign of the mean reversion parameter *α* implies that fundamentalists expect the house price to return to its fundamental value based on rents. The positive value of the return extrapolation parameter *β* means that chartists simply extrapolate previous price changes. The positive sign of *γ* implies that agents tend to switch to the better performing forecasting rule, following recent prediction performance.

The upper part of Panel B of Fig 1 shows a timeseries plot of the weight *W_t_*, the percentage of investors following the fundamentalist forecasting rule, with the scale on the right axis. The lower part of Panel B displays the distance between the actual price and the fundamental value, (*P_t_*-*F_t_*) with the scale on the left axis. In the period 1960–1980 the fundamental weight *W*_*t*_ oscillates around the 50% mark, which implies that investors are equally divided between the fundamentalist and chartist groups. A striking break in this pattern occurs in the period 1980–2007: chartists now dominate, with a weight of roughly 85 to 90%, whereas the house price rises far above its fundamental value. Eventually in the crisis years 2008–2009, however, the fundamentalist weight increases sharply and the price level falls back down.

### Endogenous dynamics

**Fig 2:**
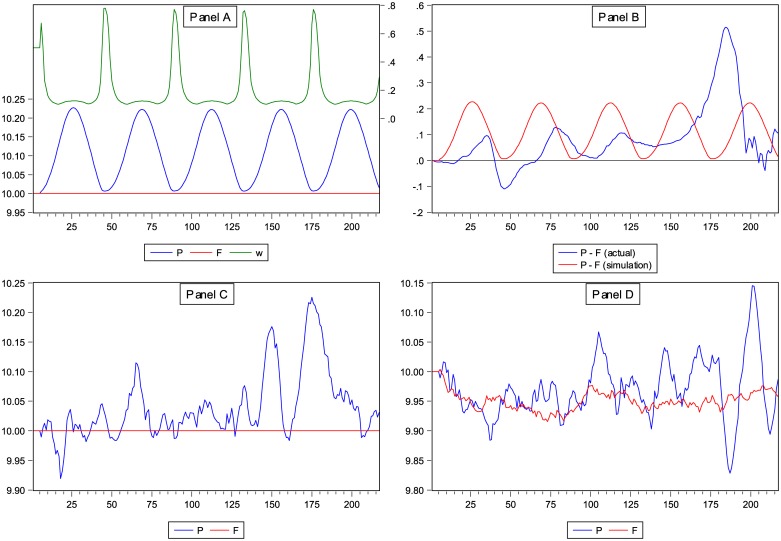
Simulated House Price Index Values. Panel A of Fig 2 displays the simulated behaviour of the log real house price index *P*_*t*_ and the proportion of investors applying the fundamentalist forecasting rule (*W*_*t*_), using the estimated model parameters. The fundamental value *F*_*t*_ is fixed at 10. In Panel B, we display both the simulated and the actual difference between *P*_*t*_ and *F*_*t*_. In Panel C we add exogenous random noise to the house price process, and in Panel D we add exogenous noise to both the price process and the fundamental value process.

We now investigate whether the multi-agent model generates house price sequences with a regular cycle, as observed in the data. For this purpose we consider Eq (13) without the stochastic error term (i.e., the deterministic part of the model), with the parameters set equal to the estimates in Table 1. For ease of exposition, the fundamental value is set at a constant value: *F_t_* = 10. Given some starting values for the prices (e.g., *P*_1_ = *P*_2_ = *P*_3_ = *P*_4_ = 10), *F*_*t*_ = 10 and *ε*_*t*+1_ = 0, we iteratively apply Eq (13) from time *t* = 5 onwards to generate a simulated sequence of house prices *P*_*t*_. Fig 2 shows the limiting behaviour of *P*_*t*_ and the fundamentalist weight *F*_*t*_ for 217 periods, the exact same number as we have actual data for.

Panel A of Fig 2 shows that the simulated price does not converge to a stable equilibrium value, as is usually the case in economic models, but to a regular boom and bust cycle repeating itself indefinitely (a stable limit cycle). Hence, there appear to be nonlinear dynamics in the U.S. housing market [16]. Prices oscillate between 10.007, just above the fundamental value, and 10.222; because these are log-prices, the house price cycle covers a non-negligible range of 21.5%. A full cycle takes 44 periods, or 11 years. Prices are pushed upwards by the real demand side of the market (coefficient *d*′) and then extrapolated by chartists. Eventually, as the mispricing *P*_*t*_-*F*_*t*_ continues to increase, the rising demand of the fundamentalists pulls the price back down again. The *increase* of the chartist weight also slows down eventually, due to the S-shaped switching function with upper bound of 1.

Panel B of Fig 2 shows the actual price deviation from fundamental value, *P*_*t*_-*F*_*t*_, versus the simulated price deviation. Both series display five peaks during the 217-period historical sample, while the amplitude and the wavelength of the simulated price cycle roughly coincide on average with the actual cycle. Apart from the one ‘negative bubble’ in the early 1970’s, the actual house price index is mostly above its fundamental value, which is also a feature of the simulated prices.

**Fig 3:**
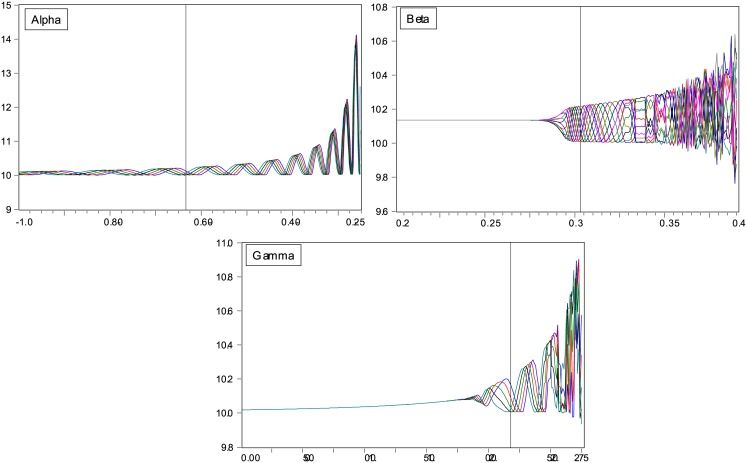
Sensitivity Analysis: Bifurcation Plots. Fig 3 displays bifurcation plots that show how the equilibrium price process changes in response to changes in the three behavioural model coefficients (*α*, *β*, and *γ*). In this sensitivity analysis we change one coefficient at a time, while keeping all other coefficients constant at their estimated values. Panel A displays the bifurcation plot for the fundamentalist coefficient *α*, Panel B the plot for the chartist coefficient *β*, and Panel C the plot for the switching parameter *γ*. The vertical lines represent the coefficient values estimated in the data.

In Panel C and D of Fig 2 we add exogenous noise to the simulation. Specifically, in Panel C we add noise to the house price process. In Panel D we add noise to both the price process and the fundamental value process. The variance of the both noise processes is set equal to the estimate in our dataset (i.e., the variance of the estimated residuals for the price process and the historical variance of the log-return of the fundamental value). Note that the noise added to the price process is the same in Panel C as in Panel D of Fig 3. The figures in Panel C and D exhibit a more realistic noisy price path compared to the smooth cycle in the deterministic simulation. The continuous boom-and-bust pattern, however, remains.

### Sensitivity analysis

As a final robustness test, we investigate the sensitivity of the limit cycle result to the value of the model coefficients. That is, we create bifurcation plots that show the sensitivity of the equilibrium price process to the value of the three behavioural coefficients in the model, *α*, *β*, and *γ*. We vary each coefficient separately over a reasonable range, while keeping the other two coefficients at their estimated values. The results are shown in Fig 3.

Panel A of Fig 3 displays the sensitivity of the equilibrium price to the fundamentalist mean-reversion coefficient *α*. The limit cycle is relatively insensitive to the value of *α*. From -1 to approximately -0.25 the model produces stable limit cycles. For lower values of *α*, the price path becomes unstable. Panel B shows the bifurcation plot for the chartist autoregressive parameter *β*. Up until *β* = 0.28, the model produces a fixed-point equilibrium. For values between 0.28 and 0.39, the solution turns into a limit cycle. Higher values result in unstable price paths. The bifurcation plot for the switching parameter *γ* in Panel C, finally, shows a stable but slowly increasing fixed point for values between zero and 1.48. Higher values give limit cycles, and even higher values non-stable patterns.

## Discussion

A multi-agent system estimated with historical U.S. house price data can endogenously generate boom-and-bust cycles, closely resembling the behavior of historical house prices. If bubbles and busts are an inherent feature of housing markets, policy makers and regulators may need to take a more active role in monitoring divergence between price levels and fundamentals, and if necessary intervene. In the words of the 2013 winner of the Nobel Prize in economic sciences, Robbert Shiller: “The sobering truth is that the current world economic crisis was substantially caused by the collapse of speculative bubbles in real estate (and stock) markets—bubbles that were made possible by widespread misunderstandings of the factors influencing prices. These misunderstandings have not been corrected, which means that the same kinds of speculative dislocations could recur”.
